# BrainChart hippocampal volume age, sex and TIV adjusted centile scores in Alzheimer’s disease

**DOI:** 10.1002/alz.086445

**Published:** 2025-01-09

**Authors:** Thomas D Parker, Richard A.I. Bethlehem, Jakob Seidlitz, Simon R White, Lena Dorfschmidt, Joshua D Bernstock, Niall Bourke, Michael CB David, Anastasia Francoise L Gailly de Taurines, Martina Del Giovane, Neil SN Graham, Magdalena A. Kolanko, Karl A Zimmerman, Paresh Malhotra, Maneesh Patel, Gregory PT Scott, Aaron Alexander‐Bloch, Edward T Bullmore, David J Sharp

**Affiliations:** ^1^ UK Dementia Research Institute Centre for Care Research and Technology, London UK; ^2^ Imperial College London, Department of Brain Sciences, London UK; ^3^ Dementia Research Centre, UCL Queen Square Institute of Neurology, University College London, London UK; ^4^ University of Cambridge, Cambridge UK; ^5^ Department of Child and Adolescent Psychiatry and Behavioral Science, The Children’s Hospital of Philadelphia, Philadelphia, PA USA; ^6^ Penn/CHOP Lifespan Brain Institute, University of Pennsylvania, Philadelphia, PA USA; ^7^ MRC Biostatistics Unit, University of Cambridge, Cambridge UK; ^8^ David H. Koch Institute for Integrative Cancer Research, Massachusetts Institute of Technology, Boston, MA USA; ^9^ Department of Neurosurgery, Brigham and Women’s Hospital, Harvard Medical School, Boston, MA USA; ^10^ King's College London, London UK; ^11^ Imperial College Healthcare NHS trust, London UK

## Abstract

**Background:**

The hippocampus is a key site of atrophy in Alzheimer’s disease (AD) and MRI derived estimates of hippocampal volume have been shown to be a robust biomarker of AD‐related neurodegeneration. However, its application at the individual level is limited by the lack of reference standards from large normative datasets that can be applied across a wide range of settings. We aimed to investigate the utility of hippocampal volume centile scores adjusted for age, sex and total intracranial volume (TIV) derived from a normative data from 101,457 participants across the life course, as a biomarker of neurodegeneration in AD.

**Methods:**

BrainChart calculates age and sex adjusted centile scores for structural MRI metrics using a GAMLSS (Generalised Additive Models for Location Scale and Shape) approach and can be applied to individual datasets using an out‐of‐sample centile scoring approach that calculates study‐specific offsets (Bethlehem, Seidlitz, White et al. Nature 2022). Using this approach, we developed models for Freesurfer derived estimates of hippocampal volume additionally incorporating TIV as a co‐variate. This was then applied to 351 individuals with pathologically confirmed AD (median scan time prior to death 5.9 years) from the NACC dataset, as well as 110 amyloid‐PET positive patients from the ADNI‐3 cohort (MCI n=71, AD dementia n=39). Wilcoxon rank‐sum tests were used to assess differences between patient groups and cognitively normal controls from the relevant datasets. Spearman’s rank correlations were used to assess relationships with cognition (MMSE score).

**Results:**

Median centile scores were reduced in all patient groups (pathologically confirmed AD cases left = 0.13, right =0.13; amyloid‐PET positive MCI cases left=0.12, right=0.16; and amyloid‐PET positive AD dementia left=0.01, right=0.02). This was significantly different to cognitively normal controls from both the NACC dataset (n=1400, cases left=0.52, right=0.52) and ADNI‐3 dataset (n=247, cases left=0.50, right=0.50), all to a significance level of p<0.0001. Hippocampal centile scores strongly predicted MMSE score in both cohorts (Spearmans’ Rho range 0.37‐0.41, p<0.001).

**Conclusion:**

BrainChart age, sex and TIV adjusted hippocampal volume centile scores, derived from an extensive normative dataset, represent a method for objectively identifying neurodegeneration in AD that is interpretable at the individual level.

